# Supplementary material for: A Critical View of the Application of the APEX Software (Aqueous Photochemistry of Environmentally-Occurring Xenobiotics) to Predict Photoreaction Kinetics in Surface Freshwaters
Source: Molecules. 2019 Dec 18;25(1):9. doi: 10.3390/molecules25010009 (PMC7017383; doi:10.3390/molecules25010009)
Supplement: Supplementary file 1 [file molecules-25-00009-s001.zip › 3.2.4_gcc-4.4.0.zip!/3.2.4_gcc-4.4.0/3.2.4_gcc-4.4.0/doc/octave/html/interpreter/Using-the-Emacs-Info-Reader-for-Octave.html]

Using the Emacs Info Reader for Octave - Untitled


Previous: Running Octave From Within Emacs,
Up: Emacs Octave Support


---

### G.4 Using the Emacs Info Reader for Octave

You may also use the Emacs Info reader with Octave's `doc` function.
For this, the package gnuserv needs to be installed.

If gnuserv is installed, add the lines

```
     (autoload 'octave-help "octave-hlp" nil t)
     (require 'gnuserv)
     (gnuserv-start)
```

to your .emacs file.

You can use either `plain' Emacs Info or the function `octave-help`
as your Octave info reader (for help -i). In the former case,
use `info_program ("info-emacs-info")`.
The latter is perhaps more attractive because it allows to look up keys
in the indices of *several* info files related to Octave (provided
that the Emacs variable `octave-help-files` is set correctly). In
this case, use `info_program ("info-emacs-octave-help")`.

If you use Octave from within Emacs, it is best to add these settings to
your ~/.emacs-octave startup file (or the file pointed to by the
Emacs variable `inferior-octave-startup-file`).
